# Outcomes of a Laparoscopic-First Approach for a Strangulated Small Bowel Obstruction: A 12-Year Single-Center Experience

**DOI:** 10.7759/cureus.96876

**Published:** 2025-11-14

**Authors:** Hideo Kidogawa, Takahito Tagami, Takeshi Konno, Takashi Okimoto, Nobutaka Matayoshi, Toshihito Uehara, Junya Noguchi, Takatomo Yamayoshi, Shin Shinyama, Kohji Okamoto

**Affiliations:** 1 Department of Surgery, Kitakyushu City Yahata Hospital, Kitakyushu, JPN

**Keywords:** bowel necrosis, bowel resection, emergency surgery, laparoscopy, minimally invasive surgery, single-incision laparoscopic surgery, strangulated small bowel obstruction

## Abstract

Background: Small bowel obstruction (SBO) is a common surgical emergency. The role of laparoscopy in managing its strangulated form is usually not preferred by many surgeons due to technical challenges and concerns about safety. This study aimed to analyze the outcomes of a "laparoscopic-first" policy for patients with suspected strangulated SBO at a single institution.

Methods: We conducted a retrospective review of all patients who underwent a laparoscopic-first approach for suspected strangulated SBO between January 2013 and December 2024. We analyzed the conversion rate to open surgery, intraoperative findings, and postoperative outcomes for patients in whom the laparoscopic procedure was successfully completed.

Results: A total of 24 patients underwent a laparoscopic-first approach. Of these, six patients (25.0%) required conversion to open laparotomy, primarily due to difficulty in safely releasing the strangulation or poor visibility. The remaining 18 patients were successfully managed laparoscopically. Within this cohort (median age: 80.0 years), the cause of obstruction was an adhesive band in all cases (100%). Bowel resection, performed extracorporeally via the single-port site in most cases, was required in 16 patients (88.9%). The median operative time was 89 minutes. Postoperative complications (Clavien-Dindo grade ≥ II) occurred in two patients (11.1%). There was no 30-day mortality. The median postoperative hospital stay was 9.5 days.

Conclusion: A laparoscopic-first approach for selected patients with strangulated SBO is a feasible and safe strategy when performed with a low threshold for conversion. Favorable short-term outcomes with low morbidity can be achieved, even in an elderly cohort requiring a high rate of bowel resection. Prudent patient selection and experienced surgical judgment are paramount to the success of this approach.

## Introduction

Small bowel obstruction (SBO) is a common surgical emergency. Its strangulated form is a time-sensitive condition requiring immediate intervention to prevent bowel ischemia, necrosis, and subsequent life-threatening complications [1]. Historically, emergency laparotomy has been the standard treatment to allow for direct assessment of bowel viability and resection of non-viable segments.

While laparoscopy has become the standard of care for simple, non-strangulated SBO due to its well-documented benefits, its role in the setting of strangulation remains a topic of considerable debate [2,3]. Concerns regarding the difficulty of manipulating dilated, friable bowel loops, the potential risk of iatrogenic perforation and contamination, and the challenges of accurately assessing bowel perfusion under pneumoperitoneum have led many surgeons to prefer an open approach in these complex cases [4].

Despite these controversies, many institutions, including our own, have increasingly adopted a "laparoscopic-first" approach for suspected strangulated SBO. This strategy involves initiating the procedure laparoscopically with a low threshold for conversion to open surgery if significant adhesions are encountered, bowel viability is uncertain, or safe manipulation is compromised. However, comprehensive data on the overall outcomes of such a policy are scarce, as many reports focus only on successfully completed laparoscopic cases rather than the entire cohort of initial attempts.

Therefore, the primary aim of this study was to retrospectively analyze the outcomes of a laparoscopic-first approach for patients with strangulated SBO at our institution over a 12-year period. We focused on the conversion rate to open surgery, the clinical outcomes of laparoscopically completed cases, and the rate of bowel resection required within this successfully managed cohort.

## Materials and methods

Study design and patient population

We conducted a retrospective review of all patients who underwent a laparoscopic-first approach for suspected strangulated SBO at Kitakyushu City Yahata Hospital, a tertiary care center in Kitakyushu, Japan, between January 2013 and December 2024. Patients were identified from a prospectively maintained surgical database. Inclusion criteria were all adult patients (age ≥ 18 years) who were taken to the operating room for suspected strangulated SBO, where laparoscopy was the intended initial approach. Exclusion criteria were patients who were planned for primary open laparotomy, those with a primary diagnosis of large bowel obstruction, or those with non-obstructive mesenteric ischemia. The primary analysis of operative details and postoperative outcomes focused on patients in whom the procedure was successfully completed laparoscopically. The rate and reasons for conversion to open surgery were also analyzed.

This study was conducted in accordance with the principles of the Declaration of Helsinki. The study protocol was approved by the Institutional Review Board (IRB) of Kitakyushu City Hospital Organization (Approval No. 202509001). The IRB waived the requirement for individual patient consent due to the retrospective nature of the study. Information regarding the study was disclosed on the hospital's website, providing patients with an opportunity to opt out.

Surgical procedure and data collection

The "laparoscopic-first approach" was the standard institutional policy for hemodynamically stable patients with suspected strangulated SBO. For this policy, stability was defined as maintaining a systolic blood pressure greater than 90 mmHg without the need for continuous vasopressor support and the absence of physical findings suggestive of diffuse peritonitis (e.g., board-like rigidity). The decision to convert to open laparotomy was made at the discretion of the attending surgeon. Common reasons for conversion included dense, inseparable adhesions; inability to safely identify and manage the point of obstruction; concerns for bowel perforation during manipulation; or hemodynamic instability.

Data were collected by reviewing patients' electronic medical records. The following variables were extracted: patient demographics (age, sex, comorbidities, American Society of Anesthesiologists (ASA) physical status classification), key preoperative laboratory values (e.g., white blood cell count, C-reactive protein, and lactate), intraoperative findings (cause of obstruction, operative time, rate of and reasons for conversion to laparotomy, rate of bowel resection), and postoperative outcomes (postoperative complications graded according to the Clavien-Dindo classification [5], length of postoperative hospital stay, and 30-day mortality).

Statistical analysis

All collected data were entered into a secure database. Descriptive statistics were used to summarize the patient and procedural characteristics. Continuous variables were presented as median and interquartile range (IQR), while categorical variables were presented as numbers and percentages. Due to the small number of patients in the non-resection group (n=2), formal statistical comparisons between the bowel resection and bowel preservation groups were not performed. The analysis is primarily descriptive. All statistical analyses were performed using R software version 4.3.2 (The R Foundation for Statistical Computing, Vienna, Austria) with the EasyR package.

## Results

Patient flow and demographics

Between January 2013 and December 2024, a total of 24 patients underwent a laparoscopic-first approach for suspected strangulated SBO. Of these, six patients (25.0%) required conversion to open laparotomy. The reasons for conversion were difficulty in safely releasing the strangulation (n=4) and poor visibility due to severe bowel distension or adhesions (n=2). For these six converted patients (median age: 81 years), the median postoperative hospital stay was 14.5 days, and postoperative complications (Clavien-Dindo grade II) occurred in two patients (33.3%). There was no 30-day mortality in this group. The remaining 18 patients were successfully managed laparoscopically and comprised the final study cohort. Individual clinical details for these 18 patients are provided in Table 1. The demographic and preoperative characteristics of these 18 patients are summarized in Table 2.

**Table 1:** Clinical characteristics and outcomes of 18 patients who underwent successful laparoscopic surgery ASA-PS: American Society of Anesthesiologists Physical Status; CRP: C-reactive protein; F: female; M: male; NA: not available; Prev abd surg: previous abdominal surgery; SSI: surgical site infection; WBC: white blood cell Note: The numerical identifiers (e.g., 1, 2, 3, etc.) used in the table are arbitrary and were created solely for the purpose of referencing specific cases within this article. These identifiers do not correspond to any patient-identifying information.

Case	Age (years)	Sex	ASA-PS	Prev abd surg	Preoperative WBC (/μL)	Preoperative CRP (mg/dL)	Preoperative lactate (mmol/L)	Cause of obstruction	Operative time (minutes)	Bowel resection	Postoperative complication (Clavien-Dindo)
1	81	F	NA	Yes	13,730	0.2	NA	Adhesive band	110	Yes	None
2	78	F	3E	Yes	18,830	1.5	1.3	Adhesive band	95	Yes	None
3	78	F	3E	No	11,700	8.52	1.9	Adhesive band	65	Yes	None
4	60	F	3E	Yes	7,000	0.08	3.9	Adhesive band	92	Yes	None
5	70	F	2E	No	9,100	0.09	1.7	Adhesive band	135	Yes	None
6	91	F	3E	Yes	14,500	0.13	3	Adhesive band	74	Yes	None
7	90	F	3E	Yes	18,000	5.54	2.5	Adhesive band	106	Yes	None
8	66	F	2E	Yes	13,000	0.65	1.8	Adhesive band	73	Yes	Ileus (I)
9	91	F	4E	Yes	8,800	1.78	NA	Adhesive band	118	Yes	None
10	62	F	2E	Yes	8,700	0.01	2	Adhesive band	96	Yes	None
11	95	M	3E	No	5,800	2.17	4.8	Adhesive band	85	Yes	None
12	68	M	3E	Yes	8,500	0.21	0.8	Adhesive band	52	No	None
13	93	F	3E	Yes	6,900	5.33	2.2	Adhesive band	96	Yes	None
14	72	M	3E	No	14,400	0.62	1.8	Adhesive band	86	Yes	SSI (I)
15	89	F	3E	No	19,200	2.8	4.3	Adhesive band	84	Yes	None
16	89	M	3E	Yes	17,700	7.37	1.2	Adhesive band	41	No	None
17	79	F	3E	No	10,600	0.1	4.1	Adhesive band	75	Yes	None
18	91	M	3E	Yes	9,600	0.49	1	Adhesive band	140	Yes	None

**Table 2:** Patient demographics and preoperative data (n=18) ASA-PS: American Society of Anesthesiologists Physical Status; CRP: C-reactive protein; IQR: interquartile range; NA: not available; WBC: white blood cell

Characteristic	Value
Age (years), median (IQR)	80.0 (70-91)
Sex, n (%)	
Male	5 (27.8%)
Female	13 (72.2%)
ASA-PS, n (%)	
2	3 (16.7%)
3	13 (72.2%)
4	1 (5.6%)
NA	1 (5.6%)	
Previous abdominal surgery, n (%)	12 (66.7%)
Laboratory values, median (IQR)	
WBC (/μL)	11150.0 (8725.0-14475.0)
CRP (mg/dL)	0.635 (0.15-2.64)
Lactate (mmol/L)	1.95 (1.6-3.23)

Illustrative case

**Figure 1:**
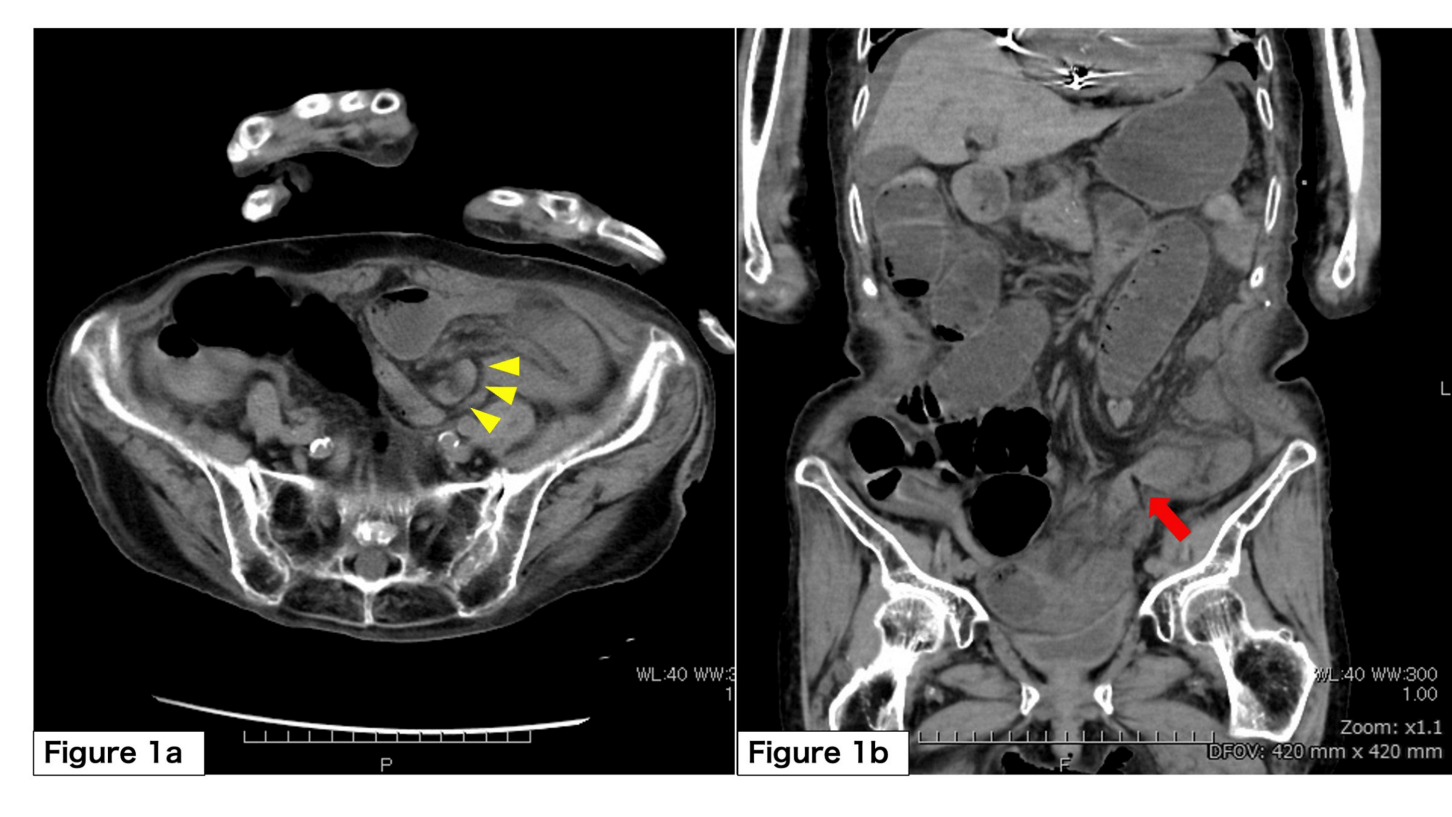
Preoperative abdominal computed tomography (CT) scan (a) Axial view showing dilated small bowel loops and mesenteric edema; (b) Coronal view demonstrating the transition point in the left lower abdomen.

A 93-year-old female resident of a nursing facility was brought to the emergency department for evaluation of abdominal pain and recurrent vomiting. Her symptoms had commenced the previous evening. Her significant past medical history included complete atrioventricular block managed with a permanent pacemaker and cognitive decline. On arrival, her vital signs were stable. Laboratory investigations revealed a normal white blood cell count (6,900/μL), a markedly elevated C-reactive protein level of 5.33 mg/dL, and a lactate level of 2.2 mmol/L. An abdominal computed tomography (CT) scan identified an SBO with a transition point in the left lower abdomen, consistent with strangulation caused by an adhesive band (Figure 1).

**Figure 2:**
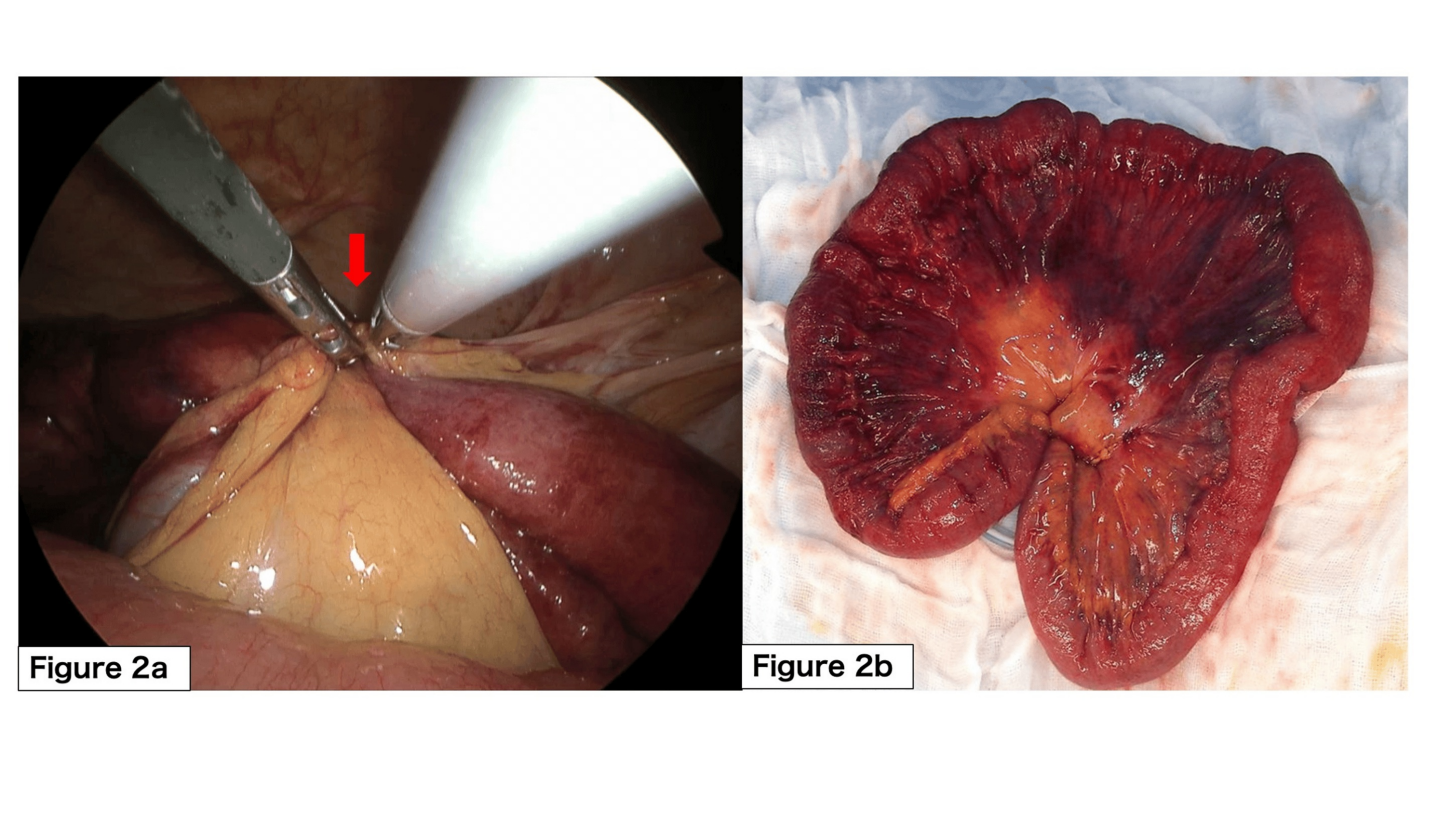
Intraoperative findings (a) Laparoscopic division of the adhesive band (red arrow) responsible for the strangulation; (b) The resected small bowel segment after exteriorization, showing dusky discoloration consistent with irreversible ischemia.

The patient was taken for emergency surgery. A 4-cm transumbilical incision was made, through which a wound retractor (Lap Protector™, HAKKO Co., Ltd., Tokyo, Japan) was placed. A multi-instrument access port (EZ Access™, HAKKO Co., Ltd.) was then mounted on the retractor to establish the single-port laparoscopy. Intraoperative exploration confirmed that a single adhesive band was causing strangulation of a small bowel segment. The bowel appeared dusky and edematous, indicating impaired blood flow and compromised viability. The constricting band was divided laparoscopically. After laparoscopic division of the constricting band, the access port was removed, and the affected bowel segment was exteriorized through the underlying wound retractor for extracorporeal resection and anastomosis under direct vision (Figure 2). The patient's postoperative course was uncomplicated. She resumed oral intake on postoperative day four and was discharged home on postoperative day 12.

Intraoperative and postoperative outcomes

The intraoperative and postoperative outcomes for the 18 laparoscopically completed cases are detailed in Table 3. The cause of obstruction was an adhesive band in all 18 patients (100%). Bowel resection was ultimately required in 16 patients (88.9%). The median operative time was 89 minutes (IQR, 74-106). 

**Table 3:** Intraoperative and postoperative outcomes (n=18) IQR: interquartile range

Outcome variable	Value
Cause of obstruction, n (%)	
Adhesive band	18 (100%)
Bowel resection, n (%)	16 (88.9%)
Operative time (minutes), median (IQR)	89 (74-106)
Postoperative complications (≥ grade II), n (%)	2 (11.1%)
Postoperative Ileus	1 (5.6%)
Surgical site infection	1 (5.6%)
Postoperative stay (days), median (IQR)	9.5 (8-13)
30-Day mortality, n (%)	0 (0%)

Postoperative complications (Clavien-Dindo grade ≥ II) occurred in two patients (11.1%), which included one case of postoperative ileus and one case of surgical site infection. No anastomotic leakage or 30-day mortality was observed. The median postoperative length of stay was 9.5 days (IQR: eight to 13 days).

## Discussion

This study demonstrates the outcomes of a "laparoscopic-first" approach for suspected strangulated SBO over a 12-year period at a single institution. Our primary findings suggest that this strategy is feasible and safe for carefully selected patients. Major international guidelines, such as the Bologna guidelines, advocate for laparoscopy in selected cases of adhesive SBO, forming the basis for such an approach [3]. We observed a 25.0% conversion rate to open laparotomy. Importantly, for the 18 patients in whom the procedure was successfully completed laparoscopically, we found favorable outcomes, including a low rate of severe complications (11.1%) and no 30-day mortality.

The 25.0% conversion rate in our series is a critical finding. It is consistent with reports specifically targeting strangulated SBO, such as the 26.5% rate reported by Kohga et al. [6], and falls within the broader range of 15%-40% reported in large meta-analyses for general adhesive SBO [4,7]. The decision to convert is multifactorial. Predictors for successful laparoscopic adhesiolysis often include fewer than two previous laparotomies, a single adhesive band, and early surgical intervention [4]. Conversely, some population-based studies have suggested that laparoscopic approaches for SBO are associated with a higher risk of iatrogenic bowel injury [8]. In this context, our conversion rate should be interpreted not as a failure but as an indicator of prudent surgical judgment. A low threshold for conversion is a crucial safety component of a laparoscopic-first policy, prioritizing patient safety over the completion of the procedure [9].

Among the laparoscopically completed cases, the outcomes were encouraging. Large-scale systematic reviews have established that for general adhesive SBO, laparoscopy is associated with lower mortality, fewer overall complications, and shorter hospital stays compared to open surgery [2,7,10]. Our study extends these findings to the specific, high-risk subgroup of strangulated SBO. Despite our cohort's high median age (80.0 years) and the high rate of bowel resection required (88.9%), our low complication rate (11.1%) and absence of mortality or anastomotic leaks suggest that the benefits of minimally invasive surgery can be achieved even in this more complex patient population. This favorable outcome in an elderly cohort may be attributed to the avoidance of the significant physiological stress associated with a full laparotomy. Elderly patients are particularly vulnerable to the major surgical trauma of open surgery, which can precipitate postoperative complications such as delirium, atelectasis, pneumonia, and prolonged ileus. These findings suggest that the benefits of a minimally invasive approach, namely reduced pain, earlier mobilization, and an attenuated systemic inflammatory response, are relatively greater in this high-risk population, contributing to the low morbidity observed in our series.

Our study also highlights a key technical aspect of modern laparoscopic SBO management: the use of laparoscopically assisted techniques. As demonstrated in our illustrative case, dividing the strangulating band laparoscopically and then exteriorizing the bowel for extracorporeal resection and anastomosis is a practical and safe hybrid technique. This approach avoids the technical challenges of a fully intracorporeal anastomosis while still minimizing the abdominal wall trauma associated with a full laparotomy [11].

This study has several limitations. First, its retrospective design is subject to inherent selection bias. Second, as a single-center experience with a small sample size, the generalizability of our findings may be limited. Third, we did not perform a comparative analysis with a primary open surgery group, which is a common limitation in studies on this topic [6]. Finally, this study focused only on short-term outcomes, and long-term data on issues such as recurrent obstruction or incisional hernia rates were not assessed [12].

## Conclusions

In conclusion, a laparoscopic-first approach is a viable and safe strategy for the management of selected patients with strangulated SBO. While a significant conversion rate must be anticipated, patients who can be successfully managed laparoscopically, even those requiring bowel resection, can demonstrate favorable short-term outcomes with low morbidity and mortality, consistent with the known benefits of minimally invasive surgery. Prudent patient selection and experienced surgical judgment are paramount to the success of this approach. This strategy should be considered a standard option in centers with laparoscopic expertise.

## References

[1] Jackson PG, Raiji MT (2011). Evaluation and management of intestinal obstruction. Am Fam Physician.

[2] Wiggins T, Markar SR, Harris A (2015). Laparoscopic adhesiolysis for acute small bowel obstruction: systematic review and pooled analysis. Surg Endosc.

[3] Ten Broek RP, Krielen P, Di Saverio S (2018). Bologna guidelines for diagnosis and management of adhesive small bowel obstruction (ASBO): 2017 update of the evidence-based guidelines from the world society of emergency surgery ASBO working group. World J Emerg Surg.

[4] Farinella E, Cirocchi R, La Mura F (2009). Feasibility of laparoscopy for small bowel obstruction. World J Emerg Surg.

[5] Dindo D, Demartines N, Clavien PA (2004). Classification of surgical complications: a new proposal with evaluation in a cohort of 6336 patients and results of a survey. Ann Surg.

[6] Kohga A, Yajima K, Okumura T, Yamashita K, Isogaki J, Suzuki K, Kawabe A (2020). Laparoscopic vs open surgery for patients with strangulated small bowel obstruction. Asian J Endosc Surg.

[7] Sajid MS, Khawaja AH, Sains P, Singh KK, Baig MK (2016). A systematic review comparing laparoscopic vs open adhesiolysis in patients with adhesional small bowel obstruction. Am J Surg.

[8] Behman R, Nathens AB, Byrne JP, Mason S, Look Hong N, Karanicolas PJ (2017). Laparoscopic surgery for adhesive small bowel obstruction is associated with a higher risk of bowel injury: a population-based analysis of 8584 patients. Ann Surg.

[9] O'Connor DB, Winter DC (2012). The role of laparoscopy in the management of acute small-bowel obstruction: a review of over 2,000 cases. Surg Endosc.

[10] Quah GS, Eslick GD, Cox MR (2019). Laparoscopic versus open surgery for adhesional small bowel obstruction: a systematic review and meta-analysis of case-control studies. Surg Endosc.

[11] Tsumura H, Ichikawa T, Murakami Y, Sueda T (2004). Laparoscopic adhesiolysis for recurrent postoperative small bowel obstruction. Hepatogastroenterology.

[12] Fevang BT, Fevang J, Lie SA, Søreide O, Svanes K, Viste A (2004). Long-term prognosis after operation for adhesive small bowel obstruction. Ann Surg.

